# SilE is an intrinsically disordered periplasmic “molecular sponge” involved in bacterial silver resistance

**DOI:** 10.1111/mmi.13399

**Published:** 2016-05-07

**Authors:** Karishma R. Asiani, Huw Williams, Louise Bird, Matthew Jenner, Mark S. Searle, Jon L. Hobman, David J. Scott, Panos Soultanas

**Affiliations:** ^1^ School of BiosciencesUniversity of NottinghamSuttonBoningtonLE12 5RDUnited Kingdom; ^2^ Centre for Biomolecular Sciences, School of Chemistry, University Park, University of NottinghamNottinghamNG7 2RDUnited Kingdom; ^3^ Oxford Protein Production Factory, Research Complex at Harwell, Rutherford Appleton LaboratoryOxfordshireOX11 0FAUnited Kingdom; ^4^ Department of ChemistryUniversity of WarwickGibbet HillCoventryCV4 7ALUnited Kingdom; ^5^ ISIS Neutron and Muon Source and Research Complex at Harwell, Rutherford Appleton LaboratoryOxfordshireOX11 0FAUnited Kingdom

## Abstract

Ag^+^ resistance was initially found on the *Salmonella enetrica* serovar Typhimurium multi‐resistance plasmid pMG101 from burns patients in 1975. The putative model of Ag^+^ resistance, encoded by the *sil* operon from pMG101, involves export of Ag^+^ via an ATPase (SilP), an effluxer complex (SilCFBA) and a periplasmic chaperon of Ag^+^ (SilE). SilE is predicted to be intrinsically disordered. We tested this hypothesis using structural and biophysical studies and show that SilE is an intrinsically disordered protein in its free *apo*‐form but folds to a compact structure upon optimal binding to six Ag^+^ ions in its *holo*‐form. Sequence analyses and site‐directed mutagenesis established the importance of histidine and methionine containing motifs for Ag^+^‐binding, and identified a nucleation core that initiates Ag^+^‐mediated folding of SilE. We conclude that SilE is a molecular sponge for absorbing metal ions.

## Introduction

Silver is a soft, shiny, lustrous and precious metal (Lansdown, 2010) with high value as a human commodity in jewellery and as an investment, and with wide applications in the electronics industry; approximately 24,000 tons of silver was mined and produced in 2012 (Mijnendonckx *et al*., 2013). Silver has also been highly valued for its broad‐spectrum antimicrobial properties and has been one of the most important antimicrobial agents prior to the discovery and introduction of antibiotics. The rapid emergence of antibiotic resistance among many bacteria has rejuvenated the interest in silver as a viable alternative antimicrobial agent (Holt and Bard, 2005; Atiyeh *et al*., 2007; Mijnendonckx *et al*., 2013).

The widespread use of silver in medical and non‐medical settings has resulted in the emergence of silver resistant bacteria. Initial reports of silver resistance date back to 1966 (Gupta *et al*., 1999; Mallard *et al*., 2012) and the first silver‐resistant plasmid pMG101, a large 180 kb plasmid assigned to the IncHI incompatibility group and reportedly carrying the *sil* operon (conferring silver resistance), was isolated from *Salmonella enterica* serovar Typhimurium following the death from septicaemia of several patients treated with silver nitrate, leading to the closure of the burn ward of the Massachusetts General Hospital (McHugh *et al*., 1975). The pMG101 *sil* resistance allowed growth of an *Escherichia coli* K‐12 (*E. coli*) strain carrying pMG101, in standard Luria‐Bertani (LB) broth containing 600 μM of Ag^+^, a concentration over six times of that known to be tolerated by *E. coli* strains K‐12 strains lacking the plasmid (Gupta *et al*., 1999).

**Figure 1:**
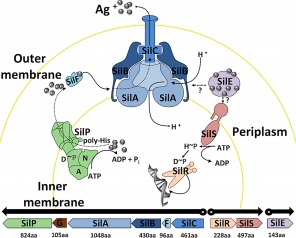
Silver resistance operon and functions of its corresponding proteins. Silver resistance proteins and their suggested active roles, deduced from homology, thought to partake in bacterial silver resistance. SilE—periplasmic metal‐binding protein, SilR and SilS—responder and membrane sensor performing two‐component transcription regulation, SilC—outer membrane protein, SilB—membrane fusion protein, SilA—chemi‐osmotic antiporter, SilP—P‐type cation ATPase and SilG (protein not depicted) and SilF—metal‐binding chaperone protein. Dashed arrows highlight the hypothetical role of SilE. The bottom line shows the mRNAs, indicating the genes and open reading frames (including number of amino acids) with the orientation of their transcription (Silver, 2003).

However, the *sil* operon comprises nine genes *sil‐PGABFCRSE* (Silver, 2003; Mijnendonckx *et al*., 2013; Hobman and Crossman, 2015; Randall *et al*., 2015), organised into three transcriptional units, *silCFBAGP*, *silRS* and *silE*, each controlled by a different promoter (Silver, 2003). The corresponding proteins have been assigned putative roles based upon homology modelling compared with other known heavy metal resistant determinants of the Pco or Cus systems (Hobman and Crossman, 2015 and Fig. 1). *silE* is under the control of its own promoter and its expression is strongly induced in the presence of Ag^+^. It codes for the 143‐amino acid long periplasmic protein SilE whose precise role in silver resistance has not been experimentally confirmed. SilE is an indispensable key component for the exogenous silver resistance phenotype (Randall *et al*., 2015), has been reported to bind between 5 and 38 Ag^+^, depending on experimental conditions (Silver *et al*., 1999; Mijnendonckx *et al*., 2013), and is often used as a marker when confirming the presence of silver resistance genes in microbes (Mirolo *et al*., 2012). It exhibits 48% sequence identity to the periplasmic copper‐binding protein PcoE which binds up to nine Cu^+^ or up to seven Ag^+^ ions (Zimmerman *et al*., 2012). The *pcoE* gene is within a cluster of seven genes (*pcoABCDRSE*) adjacent to the *sil* operon on the large *E.coli* copper resistance plasmid pRJ1004. Expression of *pcoE* is controlled by the chromosomally located copper resistance *cusRS* system (Munson *et al*., 2000). The *pco* and *sil* operons have been found together in a single locus of identical arrangement in plasmids and on the chromosomes of many Gram‐negative bacteria (Hobman and Crossman, 2015, Hao *et al*., 2015, Randall *et al*., 2015). PcoE, is believed to be unstructured in its *apo*‐form but folds and dimerizes upon Cu^+^ binding, with some α‐helical content in its secondary structure (Zimmerman *et al*., 2012). Because of its similarity to PcoE, SilE is presumed to have similar attributes as well as possess the ability to bind copper ions (Gupta *et al*., 1999; Silver *et al*., 1999; Silver, 2003; Zimmerman *et al*., 2012) although there is no experimental data available to verify its precise function. Both PcoE and SilE have ten histidine residues that are spatially conserved (Mirolo *et al*., 2012) and have been proposed to be primary candidates for metal binding (Silver, 2003; Mirolo *et al*., 2012). Following a change in environmental pH, these residues could also partake in the release of Ag^+^ into the periplasmic space with the SilCBA efflux pump ejecting the toxic monovalent metal ion, out of the cell (Mirolo *et al*., 2012). This, however, contradicts other data showing increased binding of Ag^+^ ions to SilE under acidic conditions (Silver *et al*., 1999).

In this paper, we provide experimental evidence that *apo*‐SilE is an intrinsically disordered protein (IDP) that folds to a highly α‐helical *holo*‐SilE structure upon binding to Ag^+^ ions. SilE can bind up to eight Ag^+^ ions or fewer of the harder divalent metal ions Cu^2+^ (up to six), Zn^2+^ (up to five) and Ni^2+^ (up to two), indicating a higher capacity for complexing Ag^+^ compared to other metals. We show that metal‐induced folding leads to a higher helical content with Ag^+^ followed by Cu^2+^, Zn^2+^ and Ni^2+^, consistent with its higher selectivity for Ag^+^, and confirm from mutagenesis studies that conserved histidine and methionine residues within specific sequence motifs are involved in Ag^+^ binding. We propose an Ag^+^‐mediated nucleation folding mechanism for SilE and suggest that SilE acts as a “molecular sponge” and as a first line of defence against relatively low levels of Ag^+^ ions that enter the periplasm. Potential consequences of Ag^+^‐mediated SilE folding relative to the combined bacterial silver resistance mechanism are also discussed.

## Results

### Significant primary sequence and structural features of SilE

**Figure 2:**
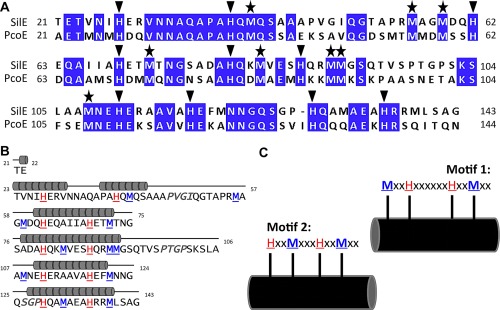
Protein sequence analysis and evaluations. A. Sequence alignment of *E. coli* PcoE with SilE from Salmonella (lacking the leader sequence, residues 1–20). The sequences, aligned using Clustal Omega (McWilliam et al., 2013) have been coloured using the “Percentage Identity” colour‐scheme in Jalview (Waterhouse et al., 2009). The sequences displaying 48% identity, the “Percentage Identity” colour‐scheme has a threshold of 80% or more being conserved residues (purple) and anything below 40% as non‐conserved (white), with the colour gradient between clarifying other less conserved residues. Conserved, aligned histidine and methionine residues have been highlighted above with either an inverted triangle or a star, respectively. B. Repeat sequence identification and secondary structure fold, predicted on Jpred can be seen above the identification of repeat sequence patterns with α‐helical predominance (six α‐helices) in the folded conformation of SilE. C. Two repeat sequence motifs (motif 1 and 2) are evident, which include the conserved histidine and methionine residues.

*S*equence alignment of native SilE and its Cu‐binding homologue PcoE was carried out using a general multipurpose primary sequence alignment program for proteins Omega (McWilliam *et al*., 2013), and amino acid sequences were coloured according to the “Percentage Identity” between the two proteins in Jalview (Waterhouse *et al*., 2009), (Fig. 2A). The calculated sequence identity between the two homologue proteins was 48% (Zimmerman *et al*., 2012). SilE and PcoE are rich in potential metal ligand‐binding histidines (ten each) and methionines (ten and fifteen, respectively). The primary sequence identity shows that the position of the ten histidine residues of the two proteins is completely conserved (Fig. 2A), suggesting a key role in metal binding.

SilE has been predicted to be an IDP. The SilE sequence was analysed through DisEMBL's and the output indicated 56% loop/coil (Supporting Information Fig. S1), suggesting that SilE has a high proportion of intrinsic disorder. In parallel, screening for possible regions of secondary structure (α‐helical, β‐strand or random coil) within the SilE (using Jpred, Cole *et al*., 2008) predicted six α‐helices across 54% of the protein sequence (Fig. 2B). Interestingly, the secondary structure predictions through Jpred are consistent with the far‐UV CD data obtained for the *holo*‐form of SilE (Supporting Information Table S1). Furthermore, two characteristic motifs, MxxHxxxxxHxxMxx (motif 1) and HxxMxxxHxxMxx (motif 2), each repeated twice within the sequence have conserved histidines and methionines that constitute potential metal‐binding motifs (Fig. 2C).

### Structural analysis of SilE with and without Ag^+^ by CD and NMR

**Figure 3:**
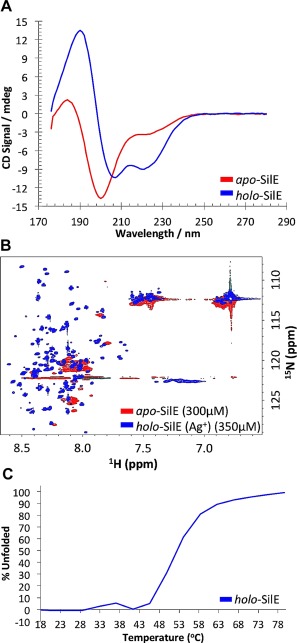
Intrinsically disordered SilE folds upon binding monovalent metal ion Ag^+^. Folding and structure content of *apo*‐SilE and *holo*‐SilE (2 mM Ag^+^), in 10 mM HEPES, 20 mM NaF, pH 7.5. A. Far‐UV CD spectrum obtained at a concentration of 57µM for *apo*‐SilE (red curve) represents very little secondary structure (negative signal around 200 nm). Upon addition of Ag^+^ (blue curve) largely negative signals are present at 207 and 221 nm typically found in proteins with significant helical structure. B. Chemical shifts seen in peaks when comparing the ^1^H/^15^N HSQC spectra of *apo*‐SilE (300 µM) (red peaks) and Ag^+^‐bound SilE (350 µM) (blue peaks), in the presence of 2 mM AgNO_3_, confirm Ag^+^‐induced folding. C. The sigmoidal temperature denaturation of *holo*‐SilE (57 µM) suggests folding in a cooperative manner.

The secondary structure of SilE was determined by far‐UV circular dichroism (CD) spectroscopy (Fig. 3A). With strong negative signals around 200 nm, the spectrum obtained for *apo*‐SilE in 10 mM HEPES, 20mM NaF, pH7.5, is typical of an unstructured, random coil polypeptide. Slight negative shoulders on the CD spectrum, at 207 and 221 nm, are consistent with a minor fraction (<20%) of α‐helical secondary structure for *apo‐*SilE (Sreerama *et al*., 1999; Greenfield, 2007; Dodero *et al*., 2011). However, these bands become considerably more prominent (∼54%) when bound to Ag^+^ in the *holo‐*SilE, with a strong negative band at 207 nm, a weaker negative ellipticity at 221 nm and a strong positive band at 190 nm (Fig. 3A), consistent with the stabilization of helical structure.

NMR spectroscopy was used to examine the tertiary structure with and without Ag^+^ (in 10 mM HEPES, 20 mM NaF, pH 7.5). The two‐dimensional ^1^H‐^15^N HSQC of *apo*‐SilE (Fig. 3B) exhibited poorly dispersed peaks, characteristic of an unfolded and unstructured protein (Dyson *et al*., 2005), and provided further experimental evidence for the largely disordered and flexible nature of SilE under native conditions. Likewise, one‐dimensional proton NMR experiments (in 10 mM sodium phosphate buffer) at several pH intervals between pH 9 and pH 5, showed no change in the local environment of *apo*‐SilE (data not shown). However, the dispersion within the NMR backbone amide ^1^H chemical shifts in the ^1^H‐^15^N HSQC spectrum of *holo‐*SilE (Fig. 3B) increased substantially in the presence of bound ${\text{Ag}}_{,}^{+}$ showing a clear signature for the induction of hydrogen bonded secondary structure.

We investigated the thermal stability of the Ag‐bound SilE complex using CD by generating a melting curve and monitoring the change in ellipticity at 207 nm. (Fig. 3C). The unfolding showed a sigmoidal transition with a mid‐point of 42°C. Moreover, the relatively sharp transition from the folded to unfolded form, (Honig *et al*., 2003), as the temperature increased suggested that only two conformational states are significantly populated.

It appears that SilE folding upon binding to Ag^+^ occurs cooperatively, as indicated by the sigmoidal thermal stability curve deduced via far‐UV CD (Fig. 3C). The monophasic, cooperative unfolding of the protein confirmed that with Ag^+^ present, the protein exists as a compact well‐folded, stable structure up to a temperature of 42°C. Moreover, the relatively sharp transition from the folded to unfolded form, characteristic of two‐state proteins (Horng *et al*., 2003), as the temperature increased suggested that only these two conformational states were present to any significant extent.

### SilE binds up to eight silver ions

**Figure 4:**
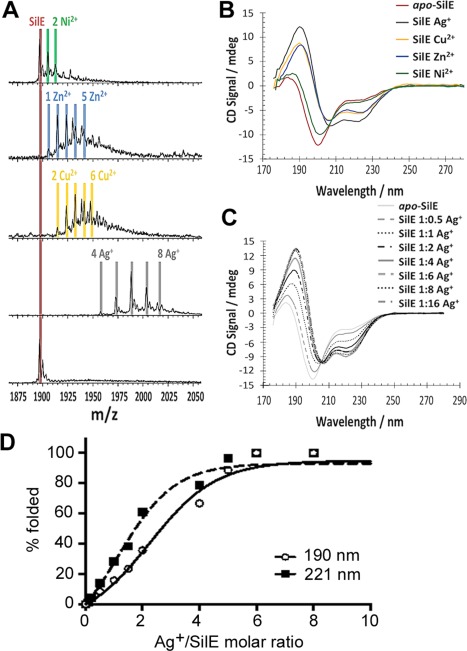
Determination of SilE metal ion‐binding. After incubation with 2 mM: Ni^2+^, Zn^2+^, Cu^2+^ and Ag^+^. A. native nano‐ESI MS for SilE (25 μM in 25 mM ammonium acetate, pH 7.0) showing the 7+ charge state. The digits together with the metal elemental symbol provide the number of atoms of metal ions bound to SilE (as deduced by the incremental mass increases) in the labeled peak. *Apo*‐SilE has molecular mass of 13,271 Da. B. Far‐UV circular dichroism data (57 µM in 10 mM HEPES, 20 mM NaF, pH 7.5) exhibiting more alpha‐helical protein content when SilE is in the presence of Ag^+^ over the other divalent metal ions, especially Ni^2+^. C. SilE titration at 75 µM in 10 mM HEPES, 20 mM NaF, pH 7.5, from 0 to 16 equivalents Ag^+^ using far‐UV CD. No change in signal following Ag^+^ addition beyond 6 Ag^+^ equivalents to SilE. D. Plots of the CD signals as a function of the Ag^+^:SilE molar ratios at 190 and 221 nm produce sigmoidal curves indicative of co‐operative uptake of Ag^+^ ions by SilE.

Interactions of certain metal ions with SilE have been reported using nano‐ESI‐MS (nano‐Electrospray Ionization‐Mass Spectrometry) under non‐denaturing conditions in volatile 25 mM ammonium acetate buffer at pH 7.0. Solutions of 2 mM of each metal ion: Ag^+^, Cu^2+^, Zn^2+^ and Ni^2+^ were added to 25 μM of *apo*‐SilE (molar ratio of 80:1). SilE‐metal complexes were observed, in each case with the relative bound proportions dependent on the protein's affinity and/or stoichiometry for the metal ion (Fig. 4A). SilE shows a distribution of Ag‐bound complexes with species containing 5, 6 and 7 bound Ag^+^ ions particularly abundant, with a maximum number of 8 detected. Binding of other harder divalent metal ions Cu^2+^ and Zn^2+^ was also evident, but with different binding stoichiometries (SilE:Cu^2+^ of 1:6; SilE:Zn^2+^ of 1:5, and a lower stoichiometry with Ni^2+^of SilE:Ni^2+^ of 1:2).

Complementary titrations using far‐UV CD (Fig. 4C) show no further change in secondary structure content after the addition of 6 molar equivalents of Ag^+^. Hence six Ag^+^ appears to be the optimum for full folding of the protein, however, the MS data suggest a further two Ag^+^ ions are capable of being bound by the folded protein. The Ag^+^ CD titration shows a very clear isodichroic point around 206 nm, which is consistent with predominantly two species in solution, namely the apo‐SilE and a predominant single ‘fully loaded’ Ag^+^‐bound form, rather than a heterogeneous mixture of different species with different binding stoichiometries. Furthermore, plots of the CD signals at 190 and 221 nm as a function of the Ag^+^/SilE molar ratio show some evidence for sigmoidal curves consistent with co‐operative uptake of Ag^+^ by SilE (Fig. 4D).

### Histidine and methionine residues are involved in Ag^+^‐binding

**Figure 5:**
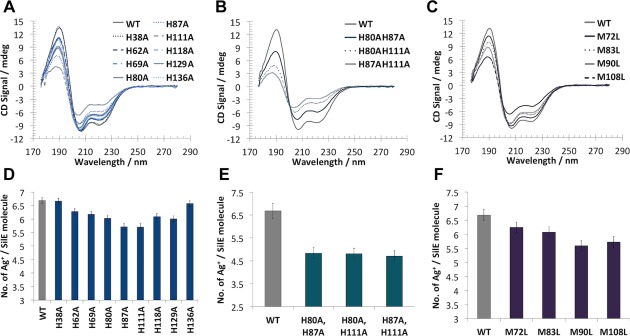
SilE histidine and methionine mutations affect protein Ag^+^ binding and folding. A–C. Far‐UV CD spectra of wild‐type *holo*‐SilE, histidine single (A) and double (B) mutants, and methionine (C) mutants with the 6 Ag^+^ equivalents at 75 μM in 10 mM HEPES, 20 mM NaF, pH 7.5. D‐F. ICP‐MS analyses of wild‐type *holo*‐SilE, histidine single (D) and double (E) mutants, and methionine (F) mutants at 50 nM in 10 mM HEPES, 20 mM NaF, pH 7.5.

In order to investigate the roles of conserved histidine and methionine residues in Ag^+^‐binding and protein folding, we mutated nine histidines to alanines (H38A, H62A, H69A, H80A, H87A, H111A, H118A, H129A and H136A) and four methionines to leucines (M72L, M83L, M90L and M108L). The secondary structure contents of all the mutant SilE proteins were measured in the presence of six Ag^+^ molar equivalents (Fig. 5A–C). H38A, H62A, H69A, H118A, H129A and H136A gave only a slight decrease in α‐helical structure (Fig. 5A and Supporting Information Table S1). In contrast, H80A, H87A and H111A exhibited large reductions in α‐helical content in comparison to wild‐type holo‐SilE (Fig. 5A and Supporting Information Table S1), implying that these residues are important for Ag^+^‐induced folding. The largest change in the CD spectra was seen with H111A which exhibits only around 60% of the wild type holo SilE secondary structure content (Supporting Information Table S1).

Based on this data, we then made three double mutant proteins, H80A/H87A, H80A/H111A and H87A/H111A. Far‐UV CD spectra of these proteins (Fig. 5B) showed a decrease in secondary structure content greater than that found with the single histidine mutant proteins with α‐helical content declining to half that of the wild type. Similar measurements for the methionine mutants found that the M108L mutant had a comparable decrease in secondary structure content to the H111A protein (Fig. 5C). The other three methionine mutants (M90L, M83L and M72L) showed effects similar to those observed for the histidine mutants (compare Fig. 5A and C).

We then measured the number of Ag^+^ bound to each of the SilE proteins using inductively coupled plasma mass spectrometry (ICP‐MS). The single histidine mutants H62A, H69A, H80A, H87A, H111A, H118A and H129A all bound on average one Ag^+^ less than the native *holo*‐SilE (Fig. 5D). Two of the single mutants, H38A and H136A did not exhibit a clear reduction in the number of bound Ag^+^ (Fig. 5D). All three double histidine mutants showed a reduction of two bound Ag^+^ (Fig. 5E) whereas all the methionine mutants showed a reduction of one bound Ag^+^ (Fig. 5F).

Collectively our data show that a number of conserved histidines and methionines are involved in Ag^+^ binding in SilE, and the ability to bind Ag^+^ has a direct effect on the holo‐SilE structure. The inability to bind Ag^+^ to key residues results in a decrease in the amount of folded protein, as judged by the reduction in CD ellipticity in the 207‐221 nm region.

### Two ‘core‐motifs’ central to the primary sequence of SilE likely form the nucleation site for Ag^+^‐induced folding

**Figure 6:**
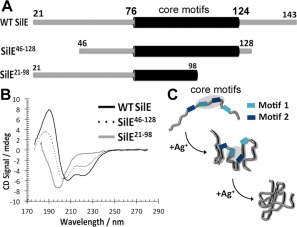
Putative core important for nucleation and Ag^+^‐induced folding of SilE. A. Schematic diagram of wild‐type (WT) SilE, after removal 20‐amino acid periplasmic signal sequence, featuring proposed putative core motifs SilE^76‐124^ (coloured black and labeled) alongside truncated polypeptides SilE^46‐128^ and SilE^21‐98^. B. Far‐UV CD spectra of wild‐type *holo*‐SilE alongside truncated mutants SilE^46‐128^ and SilE^21‐98^ with the 6 Ag^+^ equivalents at 45 μM in 10 mM HEPES, 20 mM NaF, pH 7.5. C. Schematic representation of the speculative nucleation model for Ag^+^‐mediated folding of SilE. As SilE binds Ag^+^ and following initial nucleation within the putative core motifs (highlighted) the rest of the polypeptide folds gradually to its complete *holo*‐structure upon further Ag^+^‐binding.

From our studies of mutant SilE proteins it appears that the H80A, H87A, H111A and M108L exhibited the largest folding defects upon Ag^+^ binding. These residues are located centrally within the sequence in an apparent core comprising a motif 1 (residues A77‐M91) and a motif 2 (residues E110‐F120) (Fig. 2B). Given that the secondary structure content has a high sensitivity to their mutation, we proposed that they provide initial nucleation sites for Ag^+^‐induced folding. To test this hypothesis we engineered two truncated SilE polypeptides, one SilE^46‐128^ that preserves the core region (P46 to P128) but lacks the peripheral sequences at the N‐ and C‐terminal regions, and a second SilE^21‐98^ that lacks the C‐terminal region and motif 2 from the putative nucleation core (Fig. 6A). We then studied Ag^+^‐mediated folding of these polypeptides using far‐UV CD (Fig. 6B). The SilE^46‐128^ polypeptide exhibited Ag^+^‐mediated folding similar to the wt SilE whereas the SilE^21‐98^ polypeptide did not exhibit any signs of folding upon Ag^+^ binding. These data are consistent with a model where a nucleation core is formed by central core motifs 1 and 2 which then facilitates further folding as more Ag^+^ are bound to the rest of the polypeptide.

## Discussion

Many proteins are intrinsically unstructured under physiological conditions, yet can fold when required to perform their biological functions (Dyson *et al*., 2005; Radivojac *et al*., 2007; Sibile and Bernado, 2012; Jensen *et al*., 2013; Kosol *et al*., 2013). Previous NMR studies of the SilE homologue protein PcoE have shown that it folds to a predominantly α‐helical structure upon binding ligand‐metals Cu^+^ and Ag^+^ (Zimmerman *et al*., 2012). Likewise, SilE is thought to be a putative IDP that binds to Ag^+^ but no experimental evidence was previously available to verify its structure and metal binding properties. The data presented in this paper show that that *apo*‐SilEs is an IDP lacking significant secondary structure over a range of pH conditions (Fig. 3). However, we were then able to demonstrate strong coupling between folding and metal‐uptake by showing that SilE binds Ag^+^ (Figs. 3 and 4) and folds into a moderately stable structure of high α‐helical content. Despite the involvement of multiple histidine residues in Ag^+^ binding, no pH induced folding of SilE was observed, verifying that SilE folding is specifically mediated by Ag^+^ binding.

Each SilE molecule can bind up to a maximum of eight monovalent soft metal ions of Ag^+^ in solution (Fig. 4A) but is fully folded after binding six Ag^+^: the last two Ag^+^ ions must therefore bind only to the fully folded protein. Cu^2+^, Zn^2+^ and Ni^2+^ ions, exhibit lower stoichiometries, and subsequently a lower degree of folding was seen (Fig. 4B), indicating that folding and binding are coupled and form part of the ion discrimination mechanism.

This use of folding and binding to enable uptake of a specific ion gives SilE the functional role of a ‘molecular sponge’ in the mechanism of *E. coli* silver resistance. As such its relationship to the other components in the silver resistance mechanism must be one where the unfolded protein has a high affinity for silver, but a low affinity for other cellular components. In contrast the folded protein will have a high affinity for other cellular components and as such allow SilE to off‐load its Ag^+^ to other components in the resistance mechanism. Accordingly, SilE may act either as a metal‐ion chaperone to the RND effluxer SilCBA or, as many IDPs do (Kosol *et al*., 2013) binds to or signals to the histidine kinase sensor SilS and thereby activates the remainder of the silver resistance machinery. Therefore, the the notion that SilE binds Ag^+^ and then initiates the entire mechanism of silver resistance (Sil proteins P, G, A, B, F, C, R and S, as well as positively autoregulating its own expression), by signalling or direct binding to SilS, in bacteria is a highly plausible hypothesis.

Primary sequence alignment between SilE and its homologue PcoE revealed conserved histidine and methionine residues (Fig. 2) (Mirolo *et al*., 2012) which in α‐helical structures are known to be a common feature of many metal‐binding proteins (Todd *et al*., 1991; Tanaka *et al*., 2004). We have further confirmed this to be the case in SilE by site‐specific mutagenesis. Importantly, our mutagenesis studies showed that some residues—H80, H87, H111 and M108—play a much more significant role than others in the correct Ag^+^‐mediated folding of SilE (Fig. 5). Secondary structure predictions were consistent with our experimental data, suggesting the presence of six α‐helices, however, significant helical structure was only realised in the Ag‐bound state (Fig. 2). In addition, two types of Ag^+^‐binding motifs, motif 1 MxxHxxxxxxHxxMxx and motif 2 HxxMxxxHxxMxx, where identified from the sequence in which *i* to *i+3* or *i+4* spacing between residues ensures that they appear on the same face of a folded helix. This suggests that each single helical motif in itself is unable to bind Ag^+^, but several of these together are able to (co‐operatively) co‐ordinate multiple Ag^+^ ions leading to a compact protein fold. Two of these motifs are located at the centre of the primary SilE sequence between residues S76‐G124 (Fig. 2) and we define them as the “core‐motifs” (Fig. 6). Both H80 and H87 are located in the core‐motif 2 whereas M108 and H111 are located in the core‐motif 1. We suggest the possibility of an Ag^+^‐mediated folding mechanism whereby initial Ag^+^ binding to these “core‐motifs” induces a nucleation site around which the rest of the SilE structure assembles facilitating further Ag^+^ binding (Fig. 6). This is consistent with the sigmoidal plots of CD signals at 190 and 221 nm as a function of the Ag^+^/SilE molar ratio (Fig. 4D) which are consistent co‐operative Ag^+^ binding and a folding nucleus rather than a simple sequential Ag^+^ binding and folding model. We tested this model by engineering truncated SilE polypeptides. The SilE^46‐127^ polypeptide contained both “core motifs” and exhibited Ag^+^‐mediated folding in a similar manner to the native SilE whereas the SilE^21‐97^ polypeptide lacking the core‐motif 1 exhibited no detectable Ag^+^‐mediated folding; a result consistent with our nucleation model. Our double histidine mutations H80AH87, H80H111A and H87AH111A revealed loss of two Ag^+^ ions and it is therefore likely that these pairs of histidines coordinate different Ag^+^ ions.

Complexes of Ag^+^ with histidine and imidazole can form with Ag^+^:L and Ag^+^:L_2_ stoichiometries (where L is the ligand; histidine or imidazole) with the latter being more stable, as indicated by higher stability constants and enthalpies of formation (Czoik *et al*., 2008). Ag^+^:L_2_ complexes adopt a linear geometry (Muller *et al*., 2005; Petrovac *et al*., 2012; Kumbhar *et al*., 2013) and have been proposed to coordinate Ag^+^ ions in SilE (Silver, 2003). Methionine residues are reported to co‐ordinate Ag^+^ ions in non‐linear geometries with Ag^+^:M_2_ and Ag^+^:M_3_ stoichiometries in the multicopper oxidase CueO via methionine‐rich helices (Singh *et al*., 2011) while CusF co‐ordinates Ag^+^ ions in a manner that employs histidine, methionine and tryptophan residues (Loftin *et al*., 2007). The human copper transporter 1 (hCtr1) protein in turn co‐ordinates Cu^+^ and Ag^+^ ions via histidine, methionine and cysteine residues (Du *et al*., 2013; Zhu *et al*., 2014; Rubino *et al*., 2015). It is clear that coordination of Ag^+^ ions by metal binding proteins can employ a diverse collection of amino acids and variable co‐ordination geometry. Our data collectively show that the previous theoretical model of SilE binding to five Ag^+^ via ten His residues (Silver, 2003) is not correct but only a high resolution structure of the holo‐SilE will reveal the molecular details of Ag^+^ coordination in this protein.

## Experimental procedures

### Materials

Chemicals and reagents (analytical grade) were obtained from Sigma Chemical, unless otherwise stated. All buffer exchanges were completed either by centrifugal ultrafiltration through a high flux polyethersulphone membrane with molecular weight cut‐off at 5,000 Da on a Vivaspin 20 or 2 devices or, by three cycles of four‐eight hours of dialysis against the new buffer at 4°C, using dialysis membranes with molecular weight cut‐off at 3,500 Da (Spectrum Laboratories Inc.). The sources of the metal ions were always soluble and excess ions were removed via extensive dialysis or using a Vivaspin device. The metal ion salts used are as follows:‐ Ag^+^ from silver nitrate (AgNO_3_), Cu^2+^ from cupric sulfate (CuSO_4_), (Zn^2+^) from zinc chloride (ZnCl_2_) and Ni^2+^ from nickel chloride (NiCl_2_).

### Construction of expression plasmid

The DNA sequence of the gene encoding SilE, minus the 20‐amino acid peptide leader sequence, was amplified by PCR from the *E. coli* plasmid pMG101 (Gupta *et al*., 1999). The two primer sequences are: for the forward primer 5′‐ACTGAAACCGTGAATATCCATG‐3′ and for the reverse primer 5′‐GCCTGCACTGAGCATGCG‐3′. To facilitate DNA cloning and protein expression, an *Opti3CInffwd* site was incorporated in the forward primer and in the reverse primer, an *Infusion 3'* site including a stop codon were integrated. The PCR product was then built‐into the expression vector pOPINF (OPPF), comprising the coding sequence of a His‐tag, by *In‐Fusion Reactions* (Bird, 2011). The expression construct was validated to contain the correct gene sequence insert by PCR screening, using the same cloning primers.

### PCR‐based site‐directed mutagenesis

The SilE‐pOPINF construct served as a template for PCR‐based site‐directed mutagenesis. Histidine to alanine (single and double) mutations as well as the methionine to leucine mutations were generated in 5′ and 3′ DNA fragments using the following primer combinations, where mutant introducing nucleotides are shown in bold, lowercase. The forward (Fwd) primer in each case was used to generate the mutated 5′—cDNA fragment and the reverse (Rev) primer to generate the mutated 3′‐cDNA fragment, in pairs with the flanking primers SilEPpuMIF and SilEHindIIIR.

SilEH38AFwd; 5′—GGCACCTGCT**gcc**CAGATGCAGT—3′

SilEH38ARev; 5′—ACTGCATCTG**ggc**AGCAGGTGCC—3′

SilEH62AFwd; 5′—TATGGACCAG**gcc**GAACAGGCCATTATTGCTCAT—3′

SilEH62ARev; 5′—CATGAGCAATAATGGCCTGTTC**ggc**CTGGTCCAT—3′

SilEH69AFwd; 5′—CATTATTGCT**gcc**GAAACCATGACGAACGG—3′

SilEH69ARev; 5′—CCGTTCGTCATGGTTTC**ggc**AGCAATAATG—3′

SilEH80AFwd; 5′—GGCGGATGCG**gcc**CAGAAAATGG—3′

SilEH80ARev; 5′—CCATTTTCTG**ggc**CGCATCCGCC—3′

SilEH87AFwd; 5′—GGTGGAAAGT**gcc**CAGAGGATGATG—3′

SilEH87ARev; 5′—CATCATCCTCTG**ggc**ACTTTCCACC—3′

SilEH111AFwd; 5′—AATGAATGAG**gcc**GAAAGAGCTGCAGTTG—3′

SilEH111ARev; 5′—CAACTGCAGCTCTTTC**ggc**CTCATTCATT—3′

SilEH118AFwd; 5′—TGCAGTTGCC**gcc**GAATTTATGAATAACG—3′

SilEH118ARev; 5′—CGTTATTCATAAATTC**ggc**GGCAACTGCA—3′

SilEH129AFwd; 5′—GTCTGGCCCA**gcc**CAGGCCATGG—3′

SilEH129ARev; 5′—CCATGGCCTG**ggc**TGGGCCAGAC—3′

SilEH136AFwd; 5′—GGCCGAAGCG**gcc**CGTCGCATGC—3′

SilEH136ARev—5′—GCATGCGACG**ggc**CGCTTCGGCC—3′

SilEM72LFwd; 5′—TCATGAAACC**ctg**ACGAACGGGTC—3′

SilEM72LRev; 5′ – GACCCGTTCGT**cag**GGTTTCATGA—3′

SilEM83LFwd; 5′—GCACCAGAAA**ctg**GTGGAAAGTCATCAG −3′

SilEM83LRev; 5′—CTGATGACTTTCCAC**cag**TTTCTGGTGC – 3′

SilEM90LFwd; 5′—TCATCAGAGG**ctg**ATGGGAAGTCAGAC −3′

SilEM90LRev; 5′ – GTCTGACTTCCCAT**cag**CCTCTGATGA – 3′

SilEM108LFwd; 5′—ATTAGCGGCA**ctg**AATGAGCATGAAAG −3′

SilEM108LRev; 5′ – CTTTCATGCTCATT**cag**TGCCGCTAAT – 3′

SilEPpuMIF; 5′—ATTCCCCGGAGTTAATCC*gggacc*tTTAATTC −3′

SilEHindIIIR; 5′—ATCACAAACTGGTCTAGA*aagctt*TAGCCTGC −3′

The 5′ and 3′ SilE mutated fragments were used as mega primers in a PCR including the flanking primers to generate the cDNA containing the entire translated region of the mutated His‐tagged SilE. The final cDNA constructs were cloned into the *PpuMI/HindIII* site, of the prokaryotic expression vector pOPINF. The introduction of the mutations as well as the absence of undesired spontaneous mutations was confirmed by sequencing.

### Engineering of SilE truncated polypeptides

The SilE‐pOPINF construct served as a template for PCR‐based production of the truncated polypeptides SilE^46‐128^ and SilE^21‐98^, using the following primers –

SilE^46‐128^ F; 5′—ATTCCCCGGAGTTAATCCGGGACCTTTAATTC – 3′

SilE^46‐128^ R; 5′ – GCTAATGAAAGCTTCGGTTATTAAGGGGAAACGG – 3′

SilE^21‐98^ F; 5′—CGATCGGGGCCCGCCTGTCGGGATCCAGGGG – 3′

SilE^21‐98^ R; 5′ – GCGCTTCAAGCTTGGCTTATTATGGGCCAG – 3′

The final cDNA constructs were cloned into the *PpuMI/HindIII* site for SilE^21‐98^ and into the *ApaI/HindIII* site for SilE^46‐128^, of the prokaryotic expression vector pOPINF, ensuring the presence of the N‐terminal His‐tag remained. Confirmation of complete cloning of truncated polypeptides as well as the absence of undesired spontaneous mutations was confirmed by sequencing.

### Protein overexpression and purification

All SilE over‐expression plasmids (wild‐type and mutants) were maintained in *E. coli* DH5α cells and transformed into *E. coli* BL21 Star (DE3) cells (Invitrogen) for SilE wild‐type and mutant over‐expressions. Unlabelled samples of SilE were produced from cells grown on LB medium. ^15^N labelled SilE was prepared in standard minimal media supplemented with ^15^NH_4_Cl. Each litre of medium, supplemented with 50 mg carbenicillin or 100 mg ampicillin, was inoculated with an overnight culture (5‐10 mL) of the transformed *E. coli* cells. The cells were grown aerobically with vigorous shaking at 200 rpm, 37°C, to OD_600_ ∼0.6‐0.8 and isopropyl β‐D‐1‐thiogalactopyranoside (IPTG) was added at a final concentration of 1mM to induce protein expression. After overnight induction at 37°C (200 rpm) the cells were harvested by centrifugation prior to lysis by sonication (Soniprep 150) at an output frequency of 23 kHz 12 cycles of 20 seconds at an amplitude of 10 microns, followed by 30 seconds of recovery were carried out in buffer containing 50 mM Tris‐ (tris(hydroxymethyl)aminoethane) HCl, 500 mM NaCl, pH 7.5, supplemented with 100 μg/mL lysozyme and 1 mL per 20 g cells of protease inhibitor cocktail (Sigma; for use in purification of His‐tagged proteins, DMSO solution). The cell lysate was clarified by centrifugation and the supernatant contained the soluble SilE protein.

All SilE proteins were expressed with an N‐terminal His‐tag to allow purification by affinity chromatography on nickel‐chelating Sepharose (GE Healthcare) (in column binding buffer 50 mM Tris‐HCl, 500 mM NaCl, pH 7.5 with elution in 500 mM imidazole), after which they were further purified by size‐exclusion chromatography (SEC) on a pre‐equilibrated (20 mM Tris‐HCl, 200 mM NaCl, pH 7.5) Superdex 75 column (10 mm x 300 mm) (GE Healthcare). The His‐tag was then cleaved off by incubation at 4°C overnight, with 2.5 μg HRV 3C protease per 10 mL of protein sample and all forms of SilE finally were further purified by a second round of affinity chromatography on nickel‐chelating Sepharose (GE Healthcare), whereby the protein was eluted in 50 mM Tris‐HCl, 500 mM NaCl, 30 mM‐50 mM imidazole (with the histidine mutants and truncated polypeptides requiring a lower concentration imidazole), pH 7.5. The purity and identity of the SilE proteins was confirmed by; SDS‐PAGE and nano‐ESI MS, which yielded a molecular mass of 13,268 (+/‐1.6) Da for wild‐type (Suppl. Fig. S2), corresponding to the values calculated from the sequences of SilE. All the purified SilE proteins contained no detectable metals.

### Protein Concentrations

SilE lacks light absorbing tryptophan tyrosine residues and its concentration was estimated via two alternative methods. Firstly, acquiring the Brix coefficient using an Atago DD‐7 Digital Differential Refractometer allowed us to calculate the protein concentration using the following formula as explained elsewhere (Theisen *et al*., 2000):

(dndc sucrose/dndc protein sample) x 7.8883 x Brix coefficient = (0.15/0.18) x 7.8883 x Brix coefficient

Secondly, by measuring the absorbance of the peptide bond at the ultraviolet wavelength of 205 nm on a Thermo Scientific NanoDrop 2000c spectrophotometer (Scopes, 1974). Both calculations gave similar concentration values in the mg/mL range.

### Circular dichroism spectroscopy

Far‐UV CD was used to determine the secondary structure of *apo*‐ and *holo*‐SilE in solution. CD experiments were conducted at CD1 beam line at the ASTRID2 storage ring facility at Aarhus University, Aarhus, Denmark (Hertela and Hoffman, 2011). Data was acquired at 25°C from 50 μl *apo*‐ and metal ligand ion (Ag^+^, Cu^2+^, Zn^2+^, Ni^2+^) bound‐SilE protein samples at 57 or 75 μM, in a quartz suprasil cylindrical cell (Hellma type 121.000) in a 10 mM HEPES (4‐(2‐hydroxyethyl)−1‐piperazineethanesulfonic acid), 20 mM NaF buffer at pH 7.5, with either 2 mM or titrated quantities (1‐16 equiv. Ag^+^) of metal ions added to the *holo* samples. Spectra were recorded from 170 to 280 nm, with the protein sample being in a 0.05 cm path length cell; scan speed of 20 nm min‐1 and a response time of 1 s, with each spectrum representing an average of three accumulations, with an average of 15 scans per point. A scan of buffer alone was subtracted from the protein curve. Data were converted to molar CD per residue and spectra analysis was carried out by comparing the profile of the obtained curve to those illustrated and quantified in literature (Sreerama *et al*., 1999; Greenfield *et al*., 2007; Dodero *et al*., 2011). Secondary structure percentages were calculated using the DichroWeb (Lobley *et al*., 2002) interfaces analysis programme CONTINLL, which implements the locally linearised algorithm in selecting protein sets from the reference database (Provencher and Glockner, 1981; Van Stokkum *et al*., 1990; Sreerama and Woody, 2000).

The stability of *apo* and *holo*‐SilE (2 mM Ag^+^) to temperature denaturation was tested and determined by following changes in the CD spectra. The changes in the intensity of the maximal negative signal at 200 nm for *apo* and positive signal at 190 nm for *holo*‐ (2 mM Ag^+^) were recorded as a function of increasing temperature from 18 to 80°C. The temperature was gradually increased at increments of 1°C per minute and protein samples were allowed to equilibrate at each temperature, prior to recordings at intervals of 5°C. In each case, spectra were acquired from 100 µl protein samples at 57 μM. The CD data was converted to a percentage change of the maximum CD (mdeg).

### Nano‐electrospray ionisation mass spectrometry

Experiments were carried out and spectra were recorded on a SYNAPT High Definition Mass Spectrometry (HDMS) (Waters) a hybrid quadrupole ion mobility time‐of‐flight MS instrument, with travelling‐wave ion mobility (TWIM) capability, equipped with the standard z‐spray source. The instrument conditions were optimized to provide the highest relative signals for *apo*‐ and 2 mM metal ligand ion (Ag^+^, Cu^2+^, Zn^2+^, Ni^2+^) bound‐SilE complexes at a protein concentration of 25 μM, sprayed from 25 mM ammonium acetate (C_2_H_3_O_2_NH_4_), pH 7.0. The nano‐ESI capillary voltage was 1.5 kV; cone voltage, 20 V; extraction voltage, 5 V; transfer voltage, 5 V. Other settings were as follow: trap and transfer collision voltage, 6 and 5 V, respectively; backing pressure, 1.6–1.8 mbar; trap pressure, 2.1 × 10^−2^ mbar; TOF region pressure, 1.5 × 10^−6^ mbar. Instrument control as well as data processing was carried out with the Waters MassLynx 4.1 data system. All spectra were acquired in ion positive mode and the TOF analyser operated on V‐mode. Minimum smoothing and background subtraction was applied to the obtained spectra prior to analysis.

### ICP‐MS

Ag^+^ elemental analysis of 50 nM protein diluted, with and without Ag^+^ in 5 mL solutions of 10 mM HEPES, 20 mM NaF, pH 7.5, in 1% HNO_3_, was undertaken by ICP‐MS (Thermo‐Fisher Scientific iCAP‐Q; Thermo Fisher Scientific, Bremen, Germany). The instrument was run employing three operational modes, including (i) a collision‐cell (Q cell) using He with kinetic energy discrimination (He‐cell) to remove polyatomic interferences, (ii) standard mode (STD) in which the collision cell is evacuated and (iii) hydrogen mode (H_2_‐cell) in which H_2_ gas is used as the cell gas. Samples were introduced from an autosampler (Cetac ASX‐520) incorporating an ASXpress™ rapid uptake module through a PEEK nebulizer (Burgener Mira Mist). An internal standard Rh (10 µg L^−1^) in 2% trace analysis grade (Fisher Scientific, UK) HNO_3_ was introduced to the sample stream on a separate line via the ASXpress unit. External multi‐element calibration standards (Claritas‐PPT grade CLMS‐2 from SPEX Certiprep Inc., Metuchen, NJ) included Ag, Al, As, Ba, Be, Cd, Ca, Co, Cr, Cs, Cu, Fe, K, Li, Mg, Mn, Mo, Na, Ni, P, Pb, Rb, S, Se, Sr, Tl, U, V and Zn, in the range 0 – 100 µg L^−1^ (0, 20, 40, 100 µg L^−1^). A bespoke external multi‐element calibration solution (PlasmaCAL, SCP Science, France) was used to create Ca, Mg, Na and K standards in the range 0–30 mg L^−1^. Phosphorus, boron and sulphur calibration utilized in‐house standard solutions (KH_2_PO_4_, K_2_SO_4_ and H_3_BO_3_). In‐sample switching was used to measure B and P in STD mode, Se in H_2_‐cell mode and all other elements in He‐cell mode. Peak dwell times were 10 ms for the element with 150 scans per sample. Sample processing was undertaken using Qtegra™ software (Thermo‐Fisher Scientific) utilizing external cross‐calibration between pulse‐counting and analogue detector modes when required with data being acquired in µg L^−1^. Protein concentrations were measured before and after the experiment. All glassware and plasticware used for these experiments were washed with 10% nitric acid to remove contaminating metal.

### Nuclear magnetic resonance spectroscopy

One‐dimensional ^1^H nuclear magnetic resonance (NMR) spectra were taken at 25°C on an 800 MHz Bruker Avance NMR machine, using 500 μM protein in 10 mM sodium phosphate (Na_2_HPO_4_/NaH_2_PO_4_) buffer, at various pHs ranging from 5‐9. The solvent water peak was attenuated using pulsed field gradients or by pre‐saturation.

Two‐dimensional NMR based ^15^N/^1^H Heteronuclear Single Quantum Coherence (HSQC) spectra were acquired at 25°C, on an 800 MHz Bruker Avance spectrometer. Data were collected from 0.6 mL samples of 300–350 μM ^15^N labelled sample of *apo*‐ and *holo*‐Ag^+^‐bound SilE in 10 mM HEPES buffer, 20 mM NaF, 10% D_2_O buffer at pH 7.5. Due to the higher protein concentration required for NMR experiments, Ag^+^ was dialysed into the *holo* sample a concentration a final concentration of 500 mM and excess ions were removed through further dialysis using. NMR data was processed and analyzed using Topspin package (Bruker).

### Bioinformatics

Both native protein sequences (excluding their periplasm exporting leader sequence, residues 1‐20) of PcoE and SilE were aligned, using a general multipurpose alignment program for protein primary sequences – Clustal Omega, which finds the best alignment over the entire length of each sequence submitted (McWilliam *et al*., 2013). Their percentage identity was then calculated in Jalview (Waterhouse *et al*., 2009). The sequences were coloured using the “Percentage Identity” colour‐scheme in Jalview too, to clarify the sequence similarities at a more obvious intensity.

Based on the SilE sequence, Jpred (a secondary structure prediction server that incorporates the Jnet algorithm to make more accurate predictions) was used to predict α‐helices, β‐strands and random coil (Cole *et al*., 2008). Additionally, the intrinsic protein disorder predictor DisEMBL, which utilises the PDB (Protein Data Bank), was used to predict disordered loops in SilE (Linding *et al*., 2003).

## Supporting information

Supporting Information
