# Supplementary material for: SilE is an intrinsically disordered periplasmic “molecular sponge” involved in bacterial silver resistance
Source: Mol Microbiol. 2016 May 7;101(5):731–42. doi: 10.1111/mmi.13399 (PMC5008109; doi:10.1111/mmi.13399)
Supplement: Supplementary file 1 — Supporting Information [file MMI-101-731-s001.docx]

**Supplementary Information**

**SilE is an intrinsically disordered periplasmic ‘molecular sponge’ involved in bacterial silver resistance**

Karishma R. Asiani^[a,b]^, Huw Williams^[b]^, Louise Bird^[c]^, Matthew Jenner^[d]^, Mark S. Searle^[b]^, Jon L. Hobman*^[a]^, David J. Scott^*^ ^[a,e]^, and Panos Soultanas*^[b]^

^[a]^ School of Biosciences, University of Nottingham, Sutton Bonington LE12 5RD, United Kingdom

^[b]^ Centre for Biomolecular Sciences, School of Chemistry, University Park, University of Nottingham, Nottingham NG7 2RD, United Kingdom

^[c]^ Oxford Protein Production Factory, Research Complex at Harwell, Rutherford Appleton Laboratory, Oxfordshire OX11 0FA, United Kingdom

^[d]^ Department of Chemistry, University of Warwick, Gibbet Hill, Coventry CV4 7AL, United Kingdom

^[e]^ ISIS Neutron and Muon Source and Research Complex at Harwell, Rutherford Appleton Laboratory, Oxfordshire OX11 0FA, United Kingdom

*Joint corresponding authors

Panos.soultanas@nottingham.ac.uk

David.Scott@nottingham.ac.uk

[Jon.Hobman@nottingham.ac.uk](mailto:Jon.Hobman@nottingham.ac.uk)

**Supplementary Table S1**

**
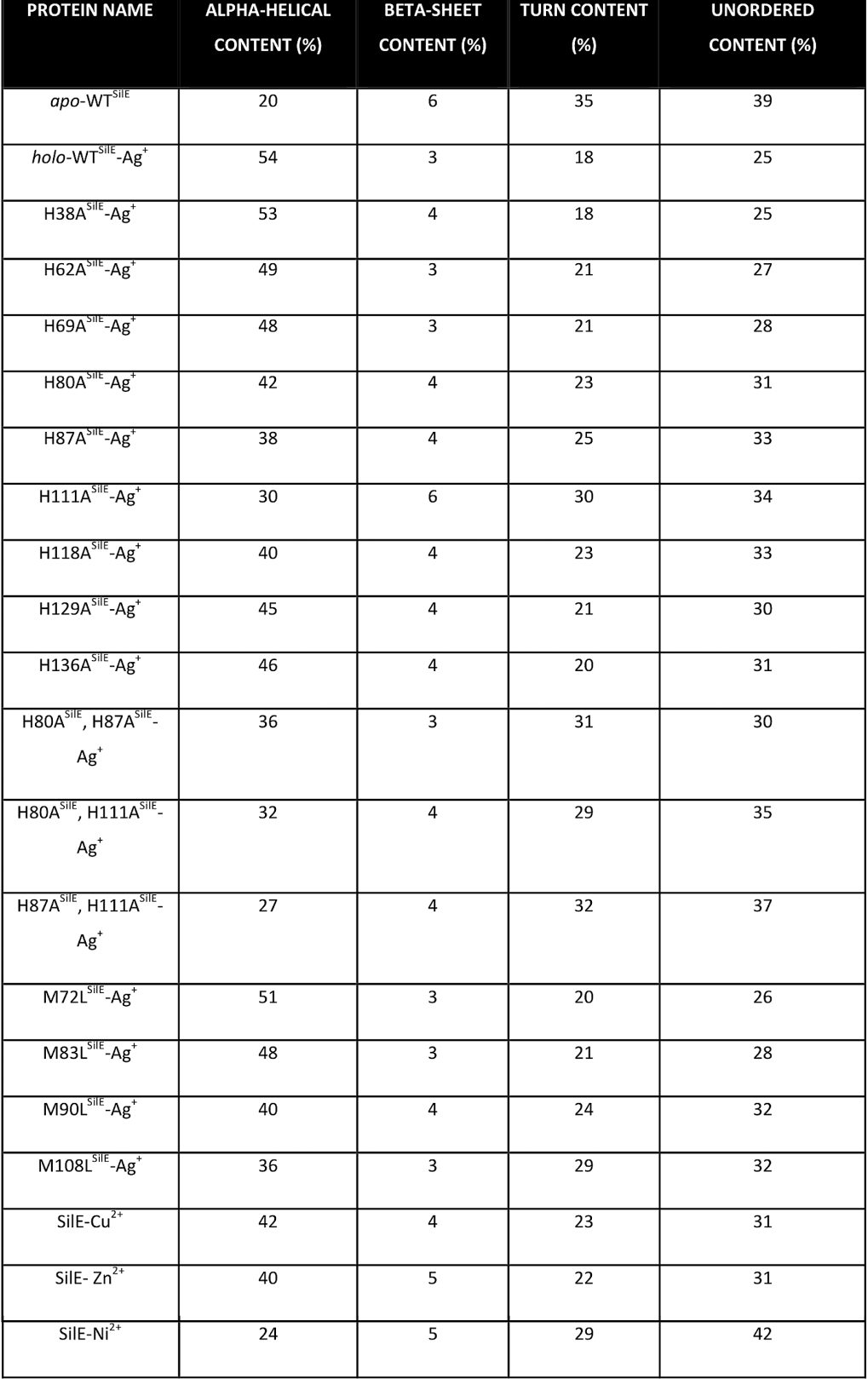
**

**Percentages of secondary structure calculated from far-UV CD data.**

CONTINLL was used via DichroWeb to calculate the percentage of secondary structure content in wild-type SilE, both in and without the presence of metal ligand ions Ag^+^, Cu^2+^, Zn^2+^, Ni^2+^ and in all mutant SilE proteins with Ag^+^.

**Supplementary Figure S1**

**
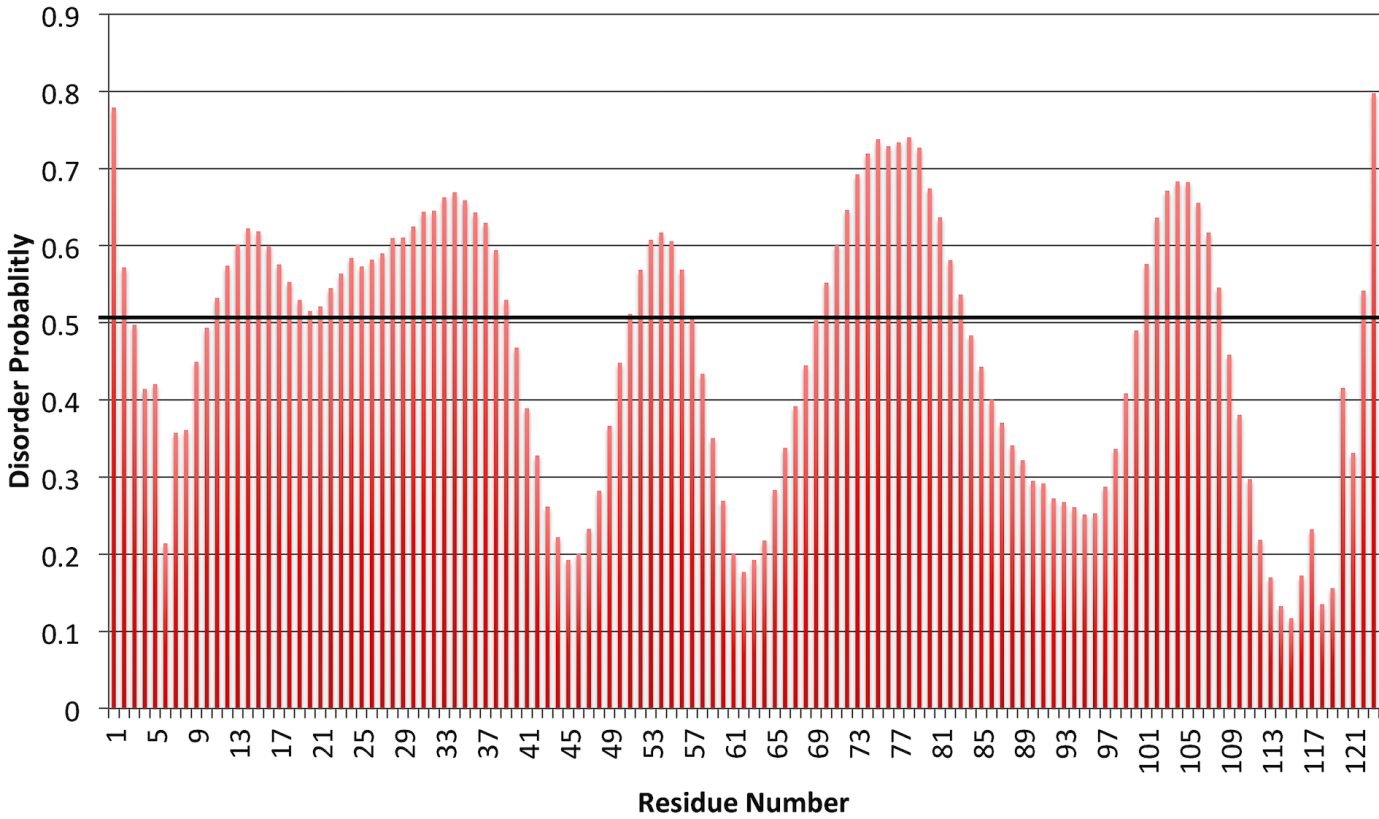
**

**DisEMBL output of SilE.**

IDP analysis using DisEMBL shows SilE is an IDP with 56% of the protein conforming to loop-coil, at a threshold of 0.516.

**Supplementary Figure S2**


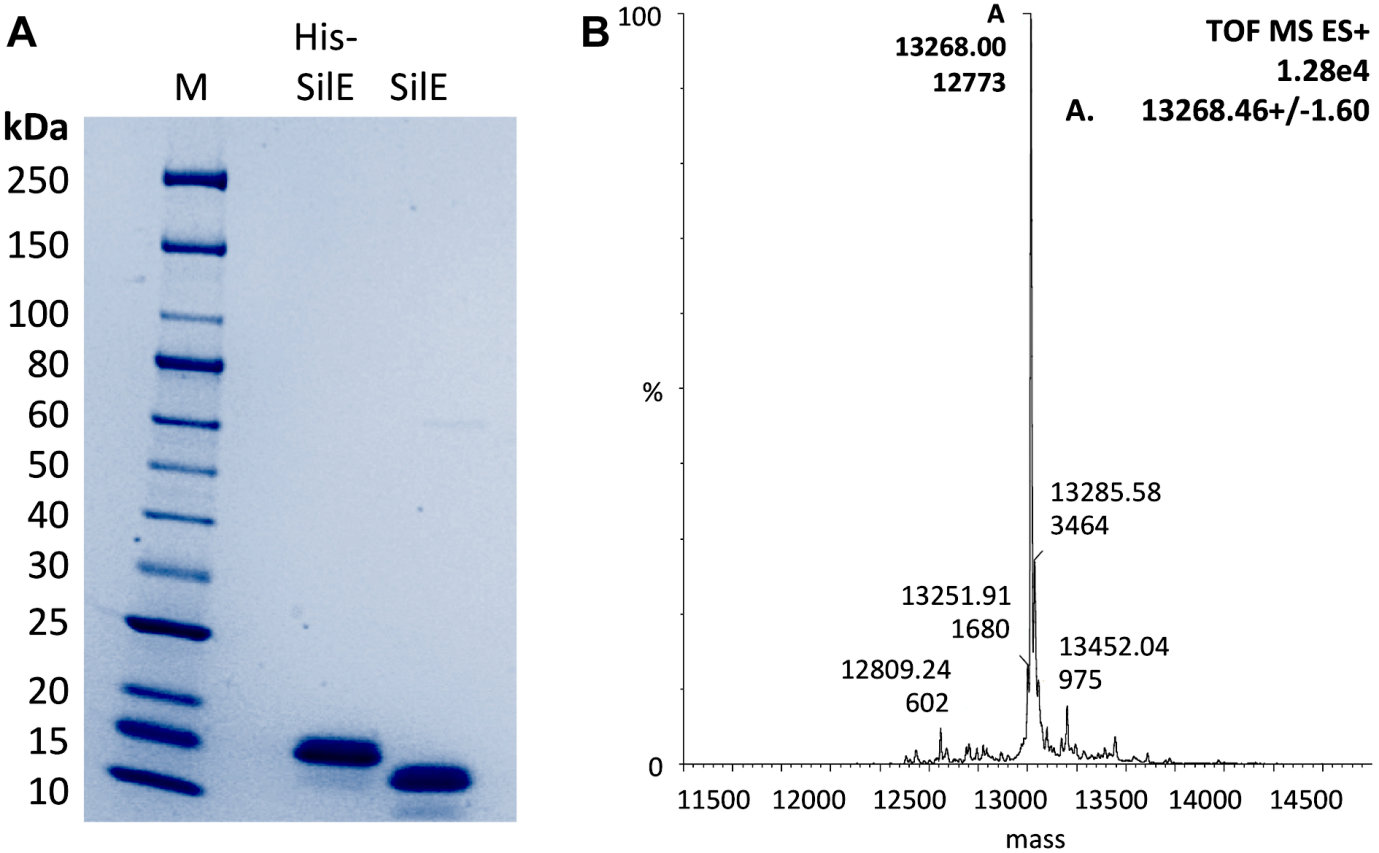


**Pure SilE**

**A** SDS-PAGE of pre His-tag cleaved and pure SilE at 13.3kDa. **B** Mass spectrometry data showing pure wild-type SilE in solution at 13,268 Da. The purity of all mutants and truncations was assessed in the same manner.
